# Heterologous booster vaccination enhances antibody responses to SARS-CoV-2 by improving Tfh function and increasing B-cell clonotype SHM frequency

**DOI:** 10.3389/fimmu.2024.1406138

**Published:** 2024-06-21

**Authors:** Yanli Song, Jiaolei Wang, Zhihui Yang, Qian He, Chunting Bao, Ying Xie, Yufang Sun, Shuyan Li, Yaru Quan, Huijie Yang, Changgui Li

**Affiliations:** ^1^ Division of the Second Vaccines, Wuhan Institute of Biological Products Co. Ltd., Wuhan, China; ^2^ Divsion of Respiratory Virus Vaccines, National Institutes for Food and Drug Control, Beijing, China

**Keywords:** homologous, heterologous, SARS-CoV-2, Libra-seq, SHM, germinal center, Tfh cells

## Abstract

Heterologous prime-boost has broken the protective immune response bottleneck of the COVID-19 vaccines. however, the underlying mechanisms have not been fully elucidated. Here, we investigated antibody responses and explored the response of germinal center (GC) to priming with inactivated vaccines and boosting with heterologous adenoviral-vectored vaccines or homologous inactivated vaccines in mice. Antibody responses were dramatically enhanced by both boosting regimens. Heterologous immunization induced more robust GC activation, characterized by increased Tfh cell populations and enhanced helper function. Additionally, increased B-cell activation and antibody production were observed in a heterologous regimen. Libra-seq was used to compare the differences of S1-, S2- and NTD-specific B cells between homologous and heterologous vaccination, respectively. S2-specific CD19+ B cells presented increased somatic hypermutations (SHMs), which were mainly enriched in plasma cells. Moreover, a heterologous booster dose promoted the clonal expansion of B cells specific to S2 and NTD regions. In conclusion, the functional role of Tfh and B cells following SARS-CoV-2 heterologous vaccination may be important for modulating antibody responses. These findings provide new insights for the development of SARS-CoV-2 vaccines that induce more robust antibody response.

## 1 Introduction

SARS-CoV-2 vaccination has effectively reduced the prevalence of coronavirus disease 2019 (COVID-19). Several different kinds of vaccines were used during the pandemic, and these vaccines exhibited variations in immunogenicity, safety, protection efficacy and effectiveness. Interestingly, a heterologous prime-boost vaccine strategy may offer advantages over a homologous approach according to numerous recent studies across inactivated, adenoviral vector and mRNA vaccine platforms (1–4). Immunization with inactivated vaccines followed by mRNA or nonreplicative viral vector vaccines has increased the effectiveness of treatment to 87%~96.5% against symptomatic COVID-19 and 95.94%~97.67% against hospitalization (5–7). In animals, heterologous prime-boosting with an adenoviral vector or a mRNA vaccine induced a 12.8- to 51.2-fold increase in protective antibodies compared to those induced by homologous inactivated vaccination (8, 9). Heterologous boosters have appeared to exert greater vaccine protection effects than homologous boosters in both preclinical and clinical trials.

In fact, Hu and his coworkers in 1992 were among the first to employ the heterologous prime-boost immunization regimes. In their study, neutralizing antibody (NAb) titers against HIV were observed in mice primed with recombinant viral vector vaccines and boosted with subunit vaccines, but not in homologous immunized with vectored or subunit vaccines (10). Lu found that heterologous prime-boost with influenza virus vaccines could improve antibody titers 4- to 19-fold change, which may provide cross-protection against seasonal influenza virus. Also, heterologous vaccination displayed increased antibody titers in polio, malaria, tuberculosis and HSV-2 vaccines (11). It may provide a new way for those antigens that are easy to mutate, difficult to develop candidate vaccines, or induce poor immune responses with homologous vaccination. However, the mechanism that heterologous prime-boost is more effective than the “homologous” prime-boost has not been fully demonstrated, which may also limit the development of this strategy in the real world.

Studies have shown that heterologous prime-boosted with COVID-19 inactivated vaccines and adenovirus vector vaccines improve higher NAb titers. The inactivated vaccines are whole virus particles, and adenovirus vector vaccines are nonreplicated vectored vaccines expressing full-length S protein, which may induce different processes of T and B cell responses (12, 13). Immune responses may complement each other to produce higher antibody responses when prime-boosted with both. However, the mechanism remains poorly understood. When repeatedly stimulated with the same COVID-19 vaccines, T cells upregulated the expression of inhibitory signals PD-1 and LAG-3 and decreased its activity (14, 15). Also, B cells activity may decline due to low activity of T cells, which may exhaust immune cells, produce immune tolerance and decrease antibody responses (14, 15). Heterologous SARS-CoV-2 vaccination stimulates more antibody-secreting cells (ASC), T cells and cytokines to enhance humoral and cellular responses, which contribute to more cross-protection to variant strains (16). Although these studies have found some discrepancies in immune responses between heterologous and homologous vaccination, they still cannot fully explain why heterologous vaccination is more effective.

Innate immune cells activate at early stage to participate in immune defense when antigen stimulation, and then APCs present antigens to T and B cells to initiate adaptive immune response. Among them, B cells and T cells play key roles in immune response sites by secreting antibodies and adjusting immune responses. During this process, follicular helper T cells (Tfh) provide cytokines and co-stimulatory signals to promote the activation, proliferation and differentiation of B cells. Subsequently, B cells differentiate into plasma cells or memory B cells (17, 18). This intricate process occurs in germinal center (GC). Understanding the differences in GC responses between heterologous and homologous prime-boosts could potentially elucidate the mechanism behind the enhanced immune responses observed with a heterologous booster dose. In our study, the differences in antibody responses, the populations and function of B cells between heterologous and homologous regimes were analyzed. Also, B-cell specific to different subunit of spike protein was analyzed to investigate the mechanism of increased antibody responses in heterologous vaccination. The study of the mechanism will provide theoretical guidance for optimizing the existing and future vaccination strategies.

## 2 Materials and methods

### 2.1 Mice and vaccination

BALB/c mice were purchased from SPF (Beijing) Biotechnology Co., Ltd. All studies were performed in accordance with protocols approved by the Institutional Animal Care and Use Committee (IACUC) at the Beijing Institute of Biological Products Co., Ltd., Beijing, China (No. BSYYF20230130002). All mice in this paper were six- to eight-week-old female mice on a BALB/c background.

**Figure 1:**
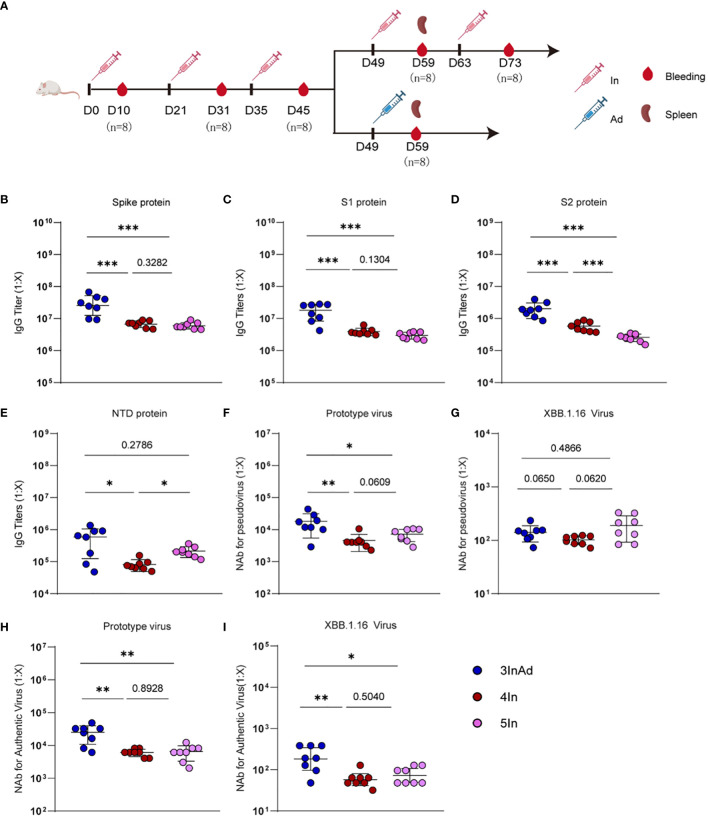
Antibody responses induced by homologous or heterologous SARS-CoV-2 vaccination in mice. **(A)** Timeline of vaccination and blood sampling. IgG titers specific to **(B)** spike, **(C)** S1, **(D)** S2, **(E)** NTD, and NAb titers against **(F)** the prototype pseudovirus, **(G)** the XBB.1.16 pseudovirus, **(H)** the prototype authentic virus, **(I)** and the XBB.1.16 authentic virus. In and Ad show inactivated vaccines and adenovirus vector vaccines, respectively. 3InAd represents mice immunized with 3 doses of In followed by one dose of Ad. 4In and 5In represent mice vaccinated with 4 or 5 identical doses of In, respectively. For **(A–I)**, n=8 per SARS-CoV-2 vaccination group, n=6 in control group. Each symbol represents one sample. The data are presented as the geometric mean ± standard error of the mean (SEM). The Mann−Whitney U test was used to analyze the differences between the indicated groups. *p < 0.05, **p < 0.01, and ***p < 0.001 were considered to indicate two-tailed significant differences. The numbers on the graph represent the p values between the indicated groups.

For the vaccination regimens, 0.5 μg prototyped SARS-CoV-2 inactivated vaccines (BBIBP-corv) combined with 22.5 μg aluminum hydroxide adjuvant (Croda) or 0.5×10^10^ viral particles (VP) of adenovirus 5 vector vaccines (Ad) encoding full-length prototyped SARS-CoV-2 spike (Convidecia) were injected intraperitoneally in 500 μL 0.01 M PBS. The control mice were vaccinated with 0.01 M PBS containing an equal concentration of aluminum hydroxide adjuvant. The first two doses were administered at D0/D21, and the subsequent booster doses were administered every 14 days (**Figure 1A**). Blood from each vaccination and spleen samples from 4In and 3InAd were collected on day 10 after vaccination (**Figure 1A**).

### 2.2 Determination of antigen-specific IgG titers in serum and culture supernatants

IgG titers specific to spike, S1 (a variable region in spike), S2 (a conserved region in spike) or NTD (a conserved region in S1) in serum and spike- or S2-specific IgG titers in supernatants were determined. 96-well plates (Costar, cat 42594) were coated with 100 μL of SARS-CoV-2 spike (Sino, cat 40589-V08H4) or S1 (Sino, cat 40591-V08B1), S2 (Sino, cat 40590-V08H1), or NTD (Sino, cat 40591-V49H) at 0.5 μg/mL overnight at 4°C. After 2 h of blocking with 3% BSA in PBS, 100 μL of each diluted serum sample or culture supernatant was incubated for 2 h at room temperature. The serum dilutions used were 1:10000 and 1:40000 (for spike, S1 and S2) and 1:1000 and 1:4000 (for NTD). The supernatants were not diluted. Standard curves were obtained by serial dilution of standard serums via titration, as previously described (19). The secondary antibody, anti-mouse IgG conjugated to HRP (Cytiva, cat. NA931V), was diluted 1:4000 in PBS-3% BSA and incubated for 1 h at room temperature. The reactions were developed with HRP substrate, and the absorbance was measured at 405 nm and 630 nm.

### 2.3 Determination of NAb against authentic and pseudo-viruses

NAb were determined using authentic and pseudo-SARS-CoV-2 virus, and the 50% inhibitory dilution (ID50) was defined as the serum dilution. NAb against authentic viruses (prototype and XBB.1.16 virus) were determined via a microcytopathogenic effect assay with a minimum eight-fold dilution and 2-fold serial dilutions, as previously described (20). NAb against pseudoviruses (prototype and XBB.1.16 virus) were determined with a minimum twenty-fold dilution and 3-fold serial dilutions, as previously described (12).

### 2.4 RNA sequencing

Spleens from the 3InAd, 4In and control groups were separated and stored in liquid nitrogen. All RNA extraction, library preparation, and sequencing were performed by BGI Genomics (Wuhan) Co., Ltd. DEGs were analyzed using DESeq2 (v1.4.5). KEGG and GO enrichment analyses were carried out as previously described (21, 22).

### 2.5 Antibodies and flow cytometry

The fixable viability dye eFluor™ 506 (eBioscience, cat 65–0866-14) was used to exclude dead cells according to the manufacturer’s instructions. The following antibodies were used for surface staining at 4°C for 30 min: α-TCR (BioLegend, clone H57–597), α-CD4 (BioLegend, clone RM4–5), α-PD-1 (eBioscience, clone J43), α-CXCR5 (BioLegend, clone L138D7), α-B220 (BioLegend, clone RA3–6B2), α-Gl7 (BioLegend, clone GL7), and α-CD95 (BioLegend, clone SA367H8). The data were analyzed using FlowJo v10.8 or CytExpert.

### 2.6 Magnetic isolation of B cells and CD4+ T cells

B cells and CD4+ T cells were separated from splenetic cell suspensions using magnetic isolation kits (Stemcell, cat. 19854 for B cells, cat. 19852 for CD4+ T cells) according to the manufacturer’s instructions. B and CD4+T cells were sorted to 90% purity and utilized in the subsequent experiment.

### 2.7 Sorting

CD4+CXCR5+PD-1+ Tfh cells or B220+CD95+Gl7+ GC B cells were sorted via the following steps. In brief, magnetically isolated CD4+ T or total B cells were stained with viability dye (eBioscience, cat 65–0866-14) and antibodies as above for 30 min at 4°C. Then, the cell suspensions were passed through 70-micron filters and resuspended in RPMI 1640 (Gibco, cat 11875093) supplemented with 10% FBS, 1 mM EDTA, 100 U penicillin, and 100 mg/ml streptomycin. Cell sorting was performed on an Aria III instrument.

### 2.8 Tfh and B-cell coculture *in vitro*

Isolated B cells and Tfh cells were cocultured as previously described with minor modifications (23, 24). 3×10^5/well B cells were cultured with or without 1.5×10^5/well Tfh cells in the presence of 5μg/ml spike peptide pools (GenScript, cat RP30020) in RPMI 1640 (Gibco, cat 11875093) supplemented with 10% heat-inactivated fetal bovine serum (Gibco, 16140071), 2 mM L-glutamine, 1 mM sodium pyruvate, 50 mM 2-mercaptoethanol, 100 U penicillin, and 100 mg/ml streptomycin. Round-bottom tissue culture plates were precultured with 2μg/ml anti-CD3 antibody (BD Biosciences, cat 553057) and 5μg/ml anti-IgM (Invitrogen, cat 16–5092-85) in PBS at 4°C overnight. Six days after coculture, the cells were stained with α-IgG1 (BioLegend, clone RMG1–1), α-IgG2b (BioLegend, clone RMG2b-1), and α-Gl7 (BioLegend, clone GL7). The supernatants were harvested, and the anti-spike IgG titers were assessed via ELISA.

### 2.9 Real-time PCR

CD4+T or B cells were magnetically sorted to 90% purity and used for RNA extraction. Total RNA was extracted from isolated cells using TRIzol reagent (Invitrogen, 15596026). First-strand cDNA was transcribed (Vazyme, cat R312–3), and real-time PCR was performed using universal qPCR SYBR Green Master Mix (Vazyme, cat Q711–03) according to the manufacturer’s protocols. The experiment was performed on a CFX96 instrument (Roche). The data were normalized to the housekeeping gene Gapdh and analyzed by the 2^(–△△CT) method.

The primers used for cDNA amplification were obtained from previous methods (25–28) and are listed as follows:

*Gapdh*, (forward) 5’-GTGAAGGTCGGTGTGAACGGATT-3’ and (reverse) 5’-GGAGATGATGACCCTTTTGGCTC-3’; Il21, (forward) 5’-GCCAGATCGCCTCCTGATTA-3’ and (reverse) 5’-CATGCTCACAGTGCCCCTTT-3’; *Bcl6*, (forward) 5’-CCGGCTCAATAATCTCGTGAA-3’ and (reverse) 5’-GGTGCATGTAGAGTGGTGAGTGA-3’; *Cxcr5*, (forward) 5’-ACTCCTTACCACAGTGCACCTT-3’ and (reverse) 5’-GGAAACGGGAGGTGAACCA-3’; *Cxcr4*, (forward) 5’-TCCAACAAGGAACCCTGCTTC-3’ and (reverse) 5’-TTGCCGACTATGCCAGTCAAG-3’; *Aicda*, (forward) 5’-GGCATGAGACCTACCTCTGC-3’ and (reverse) 5’-CAGGAGGTGAACCAGGTGAC-3’.

### 2.10 Libra-seq

Antigen-specific B cells were labeled and sorted as previously described with minor modifications (29, 30). In brief, biotinylated S1 (Acro, cat S1N-C82E8), S2 (Acro, cat S2N-C52E8) and NTD (Acro, cat S1D-C52E2) proteins were conjugated to two different streptavidin-fluorochrome conjugates, streptavidin-APC (BioLegend, cat 405207) and streptavidin-PE (BioLegend, cat 405203), in equimolar ratios. The cells were incubated with S1, S2, NTD, viability dye (eBioscience, cat. 65–0866-14) or α-CD19 (BioLegend, clone 1D3/CD19) at 4°C for 30 minutes. Then, the cells were sorted as above. Sorted cells were captured in droplets to generate nanoliter-scale gel beads in EMulsions (GEMs). The 5’ gene expression library and BCR library were prepared, and sequencing was performed by Abiosciences (Beijing) Co., Ltd. The mRNA sequencing data were processed using CellRanger version 7.0. R version 4.3.1 and the Seurat package (v5.0.0) were used for downstream analysis. The following marker genes were examined: Bcl2 and Itgax (CD11c) for memory B cells (31, 32); Fas and Mki67 for GC B cells; Xbp1 and Mzb1 for plasma cells, while other non-B cells were removed. The SHM frequency was calculated for each heavy chain sequence in the variable segment leading up to the CDR3.

### 2.11 Statistical analysis

Statistical analysis was performed utilizing GraphPad 8.0. The Mann−Whitney test was used and is explicitly described in the figure legends.

## 3 Results

### 3.1 Heterologous vaccination induced more robust antibody responses in mice

To compare the antibody responses induced by heterologous and homologous vaccination, we designed different immunization strategies (**Figure 1A**). Mice were vaccinated intraperitoneally with SARS-CoV-2 inactivated vaccines (In) or adenovirus 5 vector vaccines (Ad) encoding the full-length SARS-CoV-2 spike protein. The homologous vaccination regimen was 1 to 5 In doses (marked as In, 2In, 3In, 4In and 5In), and the heterologous immunization regimen was 3 doses of In followed by one dose of Ad (3InAd). Blood was collected on day 10 after the last homologous or heterologous booster dose. IgG titers specific to spike, S1 (a variable region in spike), S2 (a conserved region in spike), the N-terminal domain (NTD, a conserved region in S1), and neutralizing antibodies (NAb) against spike pseudovirus, as well as the authentic virus, were determined to explore the antibody dynamics after heterologous and homologous vaccination.

To assess the antibody responses in the homologous group, IgG and NAb titers were measured after each vaccination (**Supplementary Figure 1**). For SARS-CoV-2 vaccination groups, the spike-specific IgG titer peaked after the 3rd dose of the vaccine and remained at this level even after the following doses (**Supplementary Figure 1A**). The S1-specific antibody showed similar changes (**Supplementary Figure 1A**). Interestingly, S2-specific IgG levels peaked after the 4th dose and decreased after the 5th dose (**Supplementary Figure 1A**), and NTD-specific IgG levels consistently increased (**Supplementary Figure 1A**). These results indicate that more than 3 doses of homologous prime-boosts with In may induce higher titers of IgG specific to the conserved spike protein regions (**Supplementary Figure 1A**). Moreover, the titers of NAb against the prototype spike pseudovirus slightly increased after the 5th dose (**Figure 1F** and **Supplementary Figure 1B**), while the titers of NAb against the prototype authentic virus peaked after the 4th dose (**Figure 1H** and **Supplementary Figure 1C**). Additionally, we found that every dose can induce antibody responses to the XBB.1.16 virus (**Supplementary Figures 1B, C**).

We next compared antibody responses between heterologous and homologous vaccination (**Figure 1**). Compared with those in the 4In group, the spike-, S1-, S2-, and NTD-specific IgG levels were significantly higher in the 3InAd group (**Figures 1B-E**). Similar results were obtained for the titers of NAb against the prototype spike pseudovirus and the prototype authentic virus (**Figures 1F, H**). Compared with those in the 5In group, the spike-, S1-, and S2-specific IgG titers were also higher in the 3InAd group (**Figures 1B-D**), but there was no difference in the NTD-specific antibody titers (**Figure 1E**). Moreover, we found that the titers of NAb against the prototype pseudovirus or authentic virus were significantly higher in 3InAd (**Figures 1F, H**). Heterologous immunization showed stronger neutralizing effects on authentic XBB.1.16 virus (**Figure 1I**), although no differences were found on XBB.1.16 pseudovirus (**Figure 1G**). These data suggest that heterologous vaccination induced more robust IgG and NAb responses than homologous vaccination, and even the 5In homologous vaccination regimen could not induce the same antibody response as that induced by heterologous vaccination. In the following study, we further compared the 4In and 3InAd groups.

### 3.2 Heterologous vaccination induced a more robust GC response according to global transcriptomic analysis

To explore the heterogeneity of homologous and heterologous immunization strategies, RNA-seq was performed on the spleens from the heterologous and homologous group mice to compare discrepancies in the GC response according to gene expression profiling.

**Figure 2:**
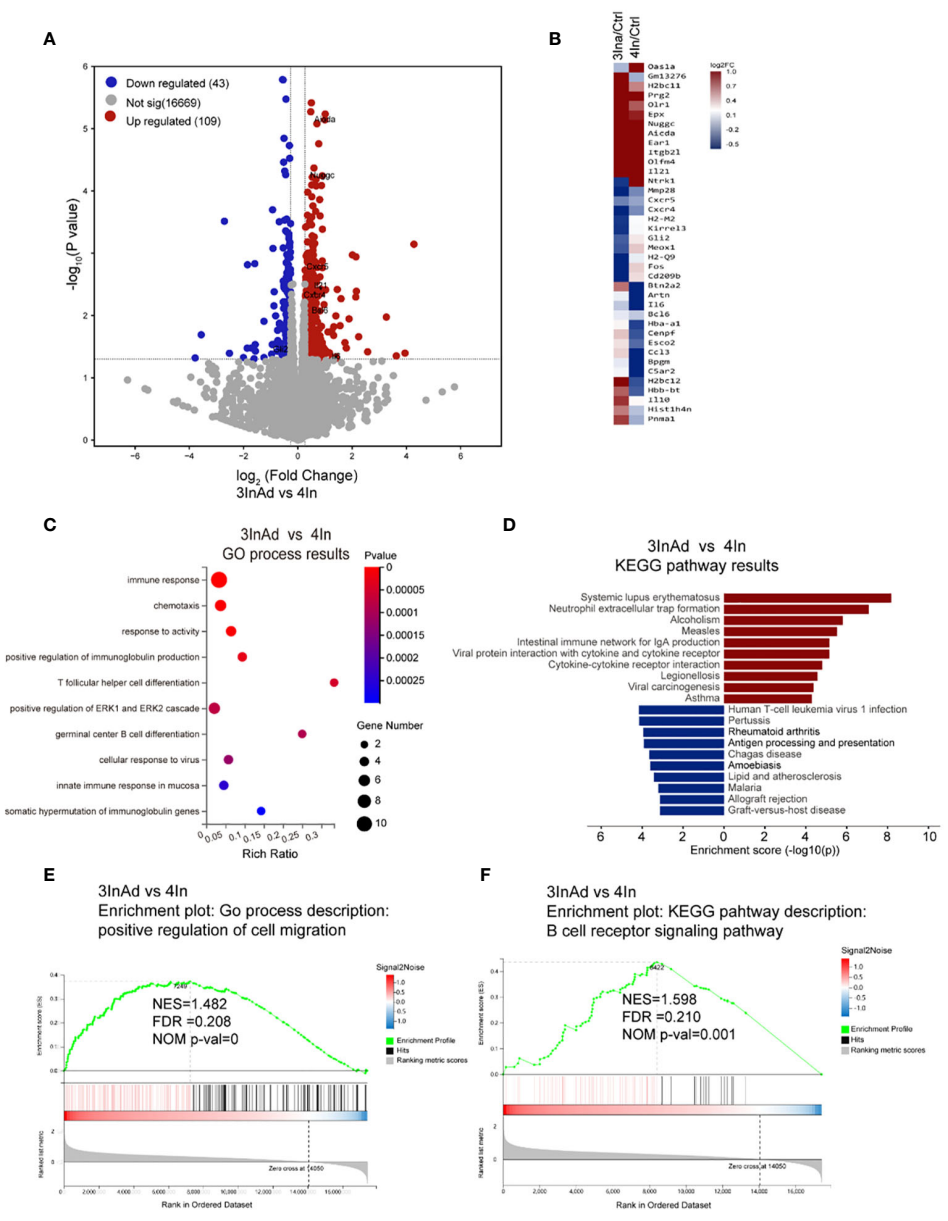
Transcriptional heterogeneity in homologous and heterologous vaccination groups. **(A)** DEGs among spleens from 3InAd *vs.* 4In. Genes with a |log_2_(fold change [FC])|>0.6 and p<0.05 were annotated as DEGs. Red dots represented upregulated genes and blue dots showed downregulated genes. **(B)** Heatmap showing the immune-related DEGs between the 3InAd and 4In groups. Four-dose control mice (Ctrl) were used to exclude background effects. **(C)** Bubble plot showing the significantly enriched BPs between the 3InAd and 4In groups, as measured by the Rich Ratio factor and q value. **(D)** Bar graph showing the significantly enriched KEGG pathways evaluated by -log_10_ (p value) in the 3InAd group compared with the 4In group. Using gene set enrichment analysis (GSEA) to analyze the expressed genes, 2 gene sets related to **(E)** cell migration and **(F)** the B-cell antigen receptor (BCR) signaling pathway were found to be significantly upregulated in the 3InAd group. NES, normalized ES; FDR, false discovery rate; NOM p-val, normalized p value. Each sample was composed of spleen samples from 3 animals. Three samples per group were used.

One hundred fifty-two differentially expressed genes (DEGs) were screened between the 3InAd and 4In groups (**Figure 2A**). Among these DEGs, 38 immune-related genes were found using the GP_CFP and Kyoto Encyclopedia of Genes and Genomes (KEGG) pathway classification methods (**Figure 2B**). Then, through GO biological process (BP) and KEGG pathway enrichment analyses, we found that these immune-related DEGs were enriched in somatic hypermutation and production of immunoglobulin (*Nuggc*, *Aicda*, *Il21*, *Il6*), Tfh differentiation (*Il21*, *Il6*), germinal center B cell differentiation (*Il6*, *Il21*), cellular response to virus (*Gli2*, *Il6*, *Il21*) (**Figures 2A, C, D**). To further explore signaling changes in the 3InAd group, we next performed gene set enrichment analysis (GSEA) using transcripts per million (TPM) values. We found that genes associated with the positive regulation of cell migration, the immunoglobulin-mediated immune response, and the positive regulation of the B-cell receptor signaling pathway were upregulated in the 3InAd group compared to the 4In group (**Figures 2E, F**). These gene expression profiling results suggest that heterologous vaccination enhances B-cell and Tfh cell responses and GC activation.

### 3.3 Heterologous immunization increased Tfh cell populations and function in GC

Tfh cells are specialized B helper cells that enable the proliferation, survival, and differentiation of GC B cells through the delivery of cytokines and costimulatory signals (12). To further investigate the changes in Tfh cells and GC responses after SARS-CoV-2 heterologous and homologous vaccination, the number and function of Tfh cells were analyzed.

**Figure 3:**
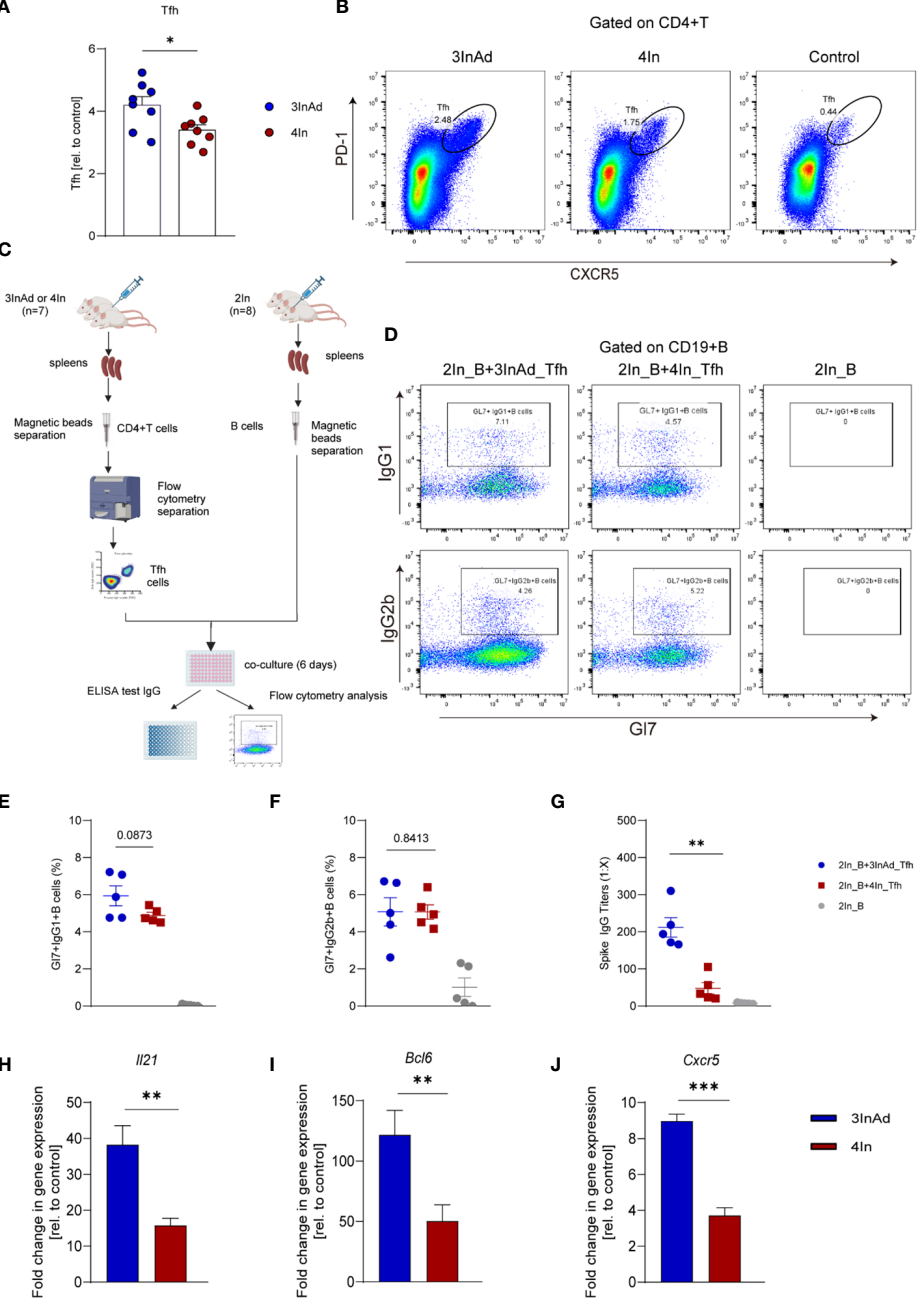
The Tfh response was enhanced in the heterologous group compared with homologous group. **(A)** Ratios of CD4+PD-1+CXCR5+ T cells (Tfh cells). Ratio = proportion of Tfh cells in the vaccine group/proportion of Tfh cells in the control group. **(B)** Representative flow plots of Tfh cells in the 3InAd and 4In groups. **(C)** Diagram of Tfh cells from heterologously vaccinated or homologously vaccinated mice cocultured with B cells isolated from mice injected with two doses of inactivated vaccine (2In). **(D–H)** Results of B-cell activation and antibody production in coculture experiments. **(D)** Representative flow plots of Gl7/IgG1 and Gl7/IgG2b gated on CD19+ B cells are shown. No plots were shown in the right panels(2In_B)because the B cells had died after being cultured *in vitro* for 6 days. **(E)** Gl7/IgG1 **(F)** and Gl7/IgG2b double-positive CD19+ B cells are shown. **(G)** Culture supernatants were harvested, and IgG titers specific to spike. **(H)** *Il21*, **(I)** *Bcl6*, **(J)** and *Cxcr5* mRNA expression were measured by qPCR. 2In_B represents B cells separated from the 2In group. 3InAd_Tfh and 4In_Tfh cells represent Tfh cells sorted from the 3InAd and 4In groups, respectively. **(A, E-J)** The data are presented as the mean ± SEM. The Mann−Whitney U test was used to analyze the differences between the indicated groups. *p < 0.05, **p < 0.01, and ***p < 0.001 were considered to indicate two-tailed significant differences. The numbers on the graph are the p values for the indicated groups. Each symbol represents one **(A)** individual animal or **(E-G)** coculture well. **(A, H-J)** n=8 in SARS-CoV-2 vaccination group, n=6 in control group. **(E-G)** n=7 mice in the 3InAd and 4In groups or n=8 mice in the 2In group.

We found that the 3InAd group showed higher Tfh cell populations than the 4In group (**Figures 3A, B**), indicating that heterologous vaccination induced more robust Tfh cell expansion. Moreover, to evaluate Tfh cell function in the heterologous group, PD1+CXCR5+ Tfh cells sorted from 3InAd or 4In group mice were cocultured with mature splenic B cells from 2In group mice (mice vaccinated with 2 doses of In), and B-cell activation defined by expression of Gl7 marker and isotype switching were detected. The coculture strategy is shown in **Figure 3C**. When B cells cocultured with 3InAd Tfh cells, we only found a slight increase of activation in the Gl7+IgG1+B group (**Figures 3D, E**), but both groups exhibit no statistical differences to the 4In Tfh cells, showing no discernible differences in Tfh helper function between heterologous and homologous groups. However, when antibodies in the coculture supernatants were further detected, a significant increase in spike-specific IgG titers was identified in B cells cocultured with 3InAd Tfh cells (**Figure 3G**), indicating an increase in IgG titers in the absence of significant B-cell activation. This discrepancy may be attributed to the different sensitivity between antibody detection and other immunologic parameters examination during the *in vitro* experiment (33). Additionally, in our study, Tfh cells were repeatedly activated by 3 doses of inactivated vaccination before the 4th-dose vaccination, therefore, the distinction of Tfh function between two groups may not be optimized.

It has been reported that IL-21, BCL-6 and CXCR5 play key roles in regulating Tfh cell differentiation and function. To compare Tfh cell proliferation and function at the gene level, we measured the mRNA expression of *Il21*, *Bcl6* and *Cxcr5* in the 3InAd and 4In groups on day 10 after vaccination. *Il21*, *Bcl6* and *Cxcr5* expression was significantly higher in the 3InAd group than that in the 4In group (**Figures 3H-J**), suggesting that enhanced Tfh cells populations and function in the heterologous group may be related to upregulating the expression of *Il21*, *Bcl6* and *Cxcr5*.

### 3.4 B-cell activation and antibody production were increased by the heterologous immunization regimen

We have proven that Tfh cell function is enhanced by heterologous vaccination. Tfh cells provide stimulatory signals to B cells, which promote their survival and ongoing proliferation (34). To compare B cells between the heterologous and homologous groups, we analyzed their proliferation and function.

**Figure 4:**
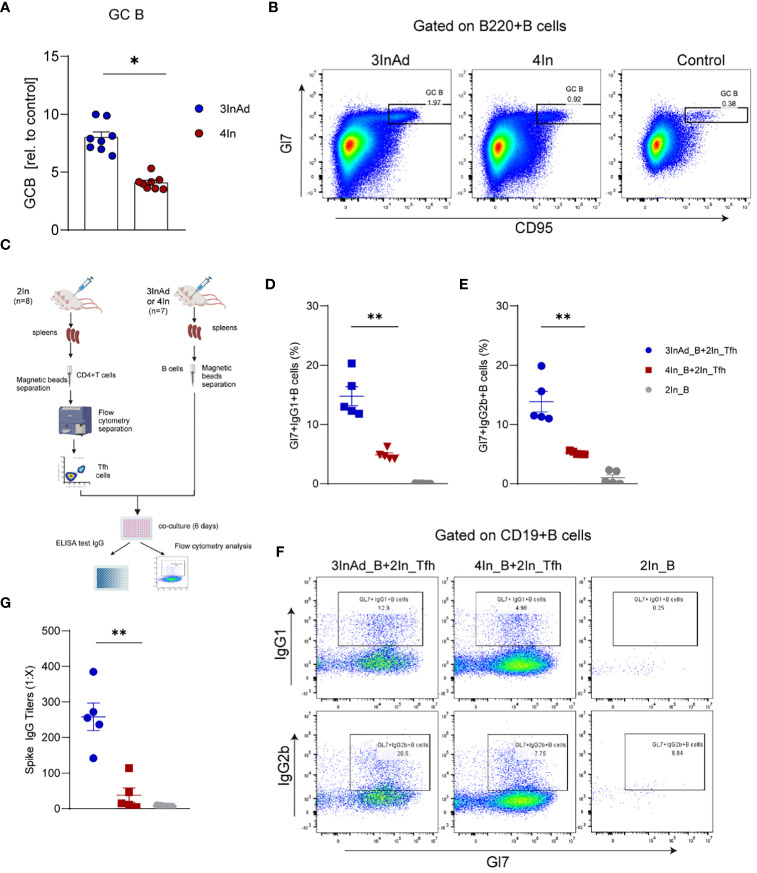
The number and function of B cells were increased in the heterologous group. **(A)** Ratios of CD95+Gl7+ B cells (GC B). Ratio = proportion of GC-B cells in the vaccine group/proportion of GC-B cells in the control group. **(B)** Representative flow plots of GC B cells gated on B220+ B cells. **(C)** Diagram of B cells from heterologously vaccinated or homologously vaccinated mice cocultured with Tfh cells isolated from the 2In group. **(D-G)** Results of B-cell activation and antibody titers via *in vitro* coculture experiments. **(D)** Gl7/IgG1 and **(E)** Gl7/IgG2b double-positive CD19+ B cells are shown. **(F)** Representative flow plots of Gl7/IgG1 and Gl7/IgG2b gated on CD19+ B cells are shown. **(G)** The culture supernatant was harvested, and IgG titers specific to spike were measured via ELISA. 3InAd_B and 4In_B represent B cells sorted from 3InAd and 4In group mice. 2In_Tfh cells represent Tfh cells sorted from the 2In group. **(A)** The data are presented as the mean ± SEM. The Mann−Whitney U test was used to analyze the differences between the indicated groups. *p < 0.05 and **p < 0.01 were considered to indicate two-tailed significant differences. The numbers in the graph represent p values. Each symbol represents **(A)** an individual animal or **(D, E, G)** one coculture well. **(A)** n=8 in SARS-CoV-2 vaccination group, n=6 in control group. **(D-G)** n=7 mice in the 3InAd and 4In groups or n=8 mice in the 2In group.

Compared to those in the 4In group, the populations of GC B cells in the 3InAd group significantly increased (**Figures 4A, B**), indicating that a heterologous boost dose promote GC B-cell expansion. Then, we further compared the activation of B-cell from these two groups. CD19+ B cells separated from the 3InAd and 4In groups were cocultured with mature splenic Tfh cells sorted from the 2In group for 6 days *in vitro* (**Figure 4C**). We found that frequencies of Gl7+IgG1+B and Gl7+IgG2b+B cells was significantly greater in the 3InAd B cells cocultured with Tfh cells than in the 4In B cell coculture (**Figures 4D-F**). Next, antibody titers in the collected coculture supernatants were measured by ELISA. We found significantly increased anti-spike IgG titers in 3InAd B cells (**Figure 4G**). These results suggest that a heterologous booster dose induces more robust B-cell activation and greater antibody production.

To assess the relationship between GC B cells and antibody responses in both the heterologous and homologous groups, Spearman correlation analysis was used. We found a strong positive correlation between spike-specific IgG titers and the percentage of GC B cells in the 3InAd and 4In groups (**Supplementary Figure 2A**). This correlation was also observed between NAb titers against authentic virus and the frequencies of GC B cells in both the heterologous and homologous groups (**Supplementary Figure 2B**). These results suggest that heterologous vaccine induced more SARS-CoV-2-specific B cells, which led to higher antibody titers.

### 3.5 A heterologous booster dose promoted higher somatic hypermutation and B-cell clonal expansion specific to conserved regions

We have demonstrated that heterologous vaccination increases B-cell activation and antibody production in mice. B cells play a vital role in immune responses by generating NAb that defend SARS-CoV-2 infection and prevent reinfection (35). To compare the differences of antigen specific B-cell responses in heterologous and homologous vaccination, Libra-seq (linking B-cell receptor to antigen specificity through sequencing) was used to map spike-, S1-, S2- and NTD-specific BCR sequences. The number of SHMs and the expansion of antigen-specific B cells were evaluated.

**Figure 5:**
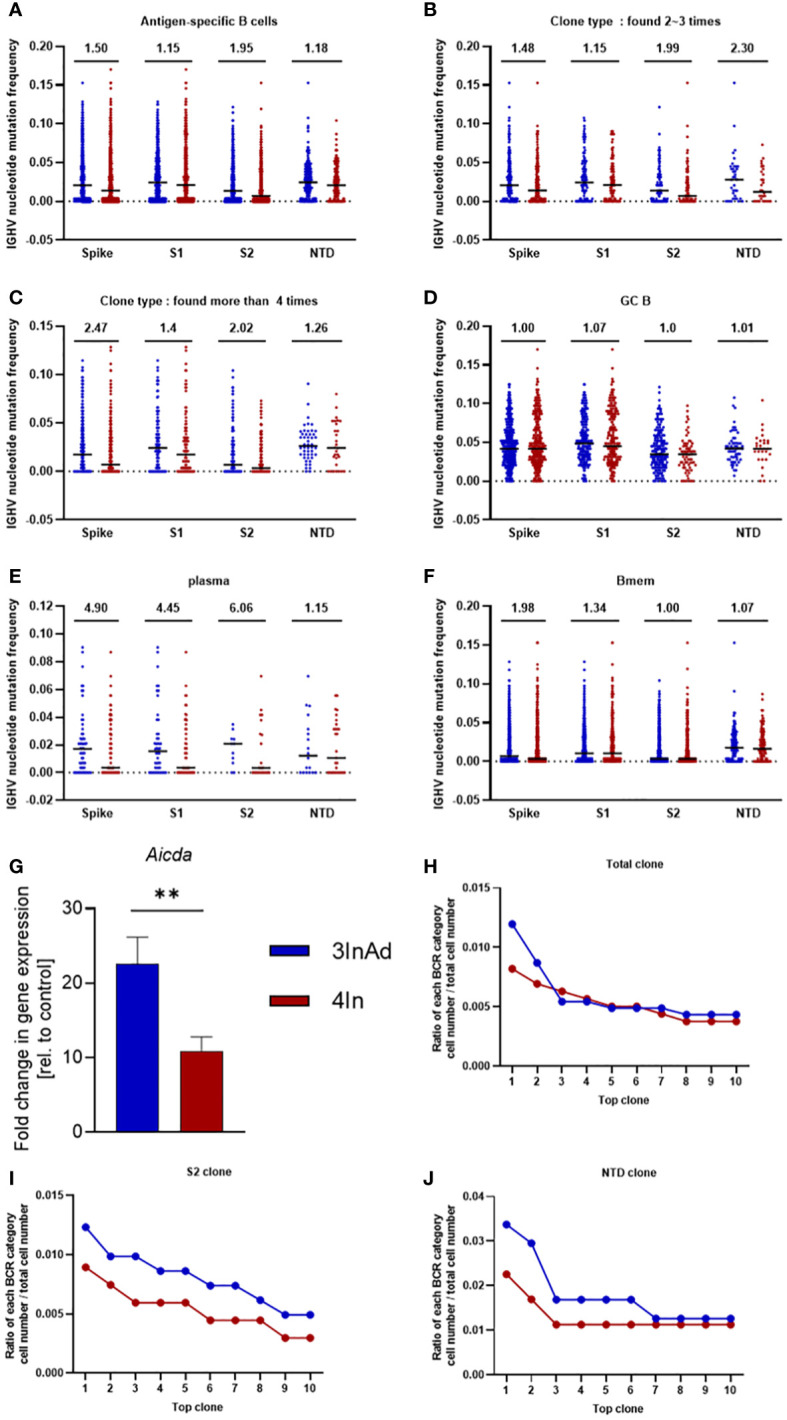
Somatic hypermutation (SHM) and clonal expansion in the heterologous vaccine group. Mutation frequency for spike-, S1-, S2- and NTD-specific **(A)** CD19+ clones; **(B)** moderately expanded clones (found 2~3 times); **(C)** highly expanded clones (found more than four times); **(D)** GC B cells; **(E)** plasma cells; and **(G)** memory B cells. **(G)** *Aicda* mRNA expression in B cells was measured by qPCR. The ratio of the top 10 BCR classifications to **(H)** spike-, **(I)** S2-, and **(J)** NTD-specific B-cell numbers. Ratio=cell count of each BCR classification/antigen-specific B-cell count. **(F)** n=8 for the SARS-CoV-2 vaccination group and n=6 for control group. The data are presented as the mean ± SEM. The Mann−Whitney U test was used to analyze the differences between the indicated groups. **p < 0.01 was considered to indicate a two-tailed significant difference. **(A–G)** The numbers in the graph represent the mutation ratio. Ratio=3InAd mutation ratio/4In mutation ratio. Each dot represents the hypermutation frequency of one B cell. One sequenced sample was formed by mixing 10 mouse spleen cells in each group to eliminate individual variances.

Compared to those in the 4In group, the SHMs in the 3InAds group were increased in spike-, S1-, S2- and NTD-specific B cells (**Figure 5A**). S2-specific B cells exhibited 1.95-fold increases, respectively (**Figure 5A**). The 3InAd group exhibited increased SHMs in highly expanded clones (found more than 4 times), moderately expanded clones (found 2 to 3 times) and singleton clones (**Figures 5B, C**, **Supplementary Figure 3A**). These results suggest that a heterologous booster dose promoted higher SHM of B cells specific to conversed regions. We next assessed the SHM in phenotypes of the B cells above in heterologous and homologous groups. There was a slight change in the SHM of GC B cells in the 3InAd group compared to the 4In group (**Figure 5D**). The SHMs in the spike-, S1-, S2- and NTD-specific plasma cells from the 3InAds group exhibited 4.90-, 4.45-, 6.06- and 1.15-fold increases, respectively (**Figure 5E**). Additionally, we observed 1.98- and 1.34-fold SHM increases in spike- and S1-specific memory B cells, respectively (**Figure 5F**). These results suggest that the increased SHM of the heterologous group was mainly enriched in S2-specific plasma cells, which may contribute to high-affinity antibodies.

SHMs are regulated by activation-induced cytidine deaminase (AID), an enzyme that induces point mutations in CDR3 and leads to B-cell expansion (36–38). It is predominantly expressed in CXCR4^hi^GC B cells^43^. To further assess the source of SHMs in the B cells of heterologous and homologous groups, we measured the mRNA expression of *Aicda* (encoded AID) and *Cxcr4* in GC B cells. We found that *Aicda* and *Cxcr4* were significantly upregulated in the 3InAds group compared to the 4Ins group (**Figure 5G** and **Supplementary Figure 3B**), indicating that heterologous vaccination upregulated higher AID expression to increased SHMs in B cells than homologous group.

To measure the expansion and proliferation of B cells, we calculated the cell number ratio of each BCR clonotype to each antigen-specific B-cell number. There was a greater percentage of spike-specific top 2 BCR clones in the 3InAd group (**Figure 5H**). Similarly, the proportions of the top 10 BCR clones specific to S1 exhibited similar tendencies in the 3InAd and 4In groups (**Supplementary Figure 3C**), whereas the proportions of the S2- and NTD-specific clones in the 3InAd group were greater than those in homologous group (**Figures 5I, J**). These data demonstrated that heterologous vaccination induced greater expansion of BCRs enriched in the conserved regions in the spike protein.

## 4 Discussion

**Figure 6:**
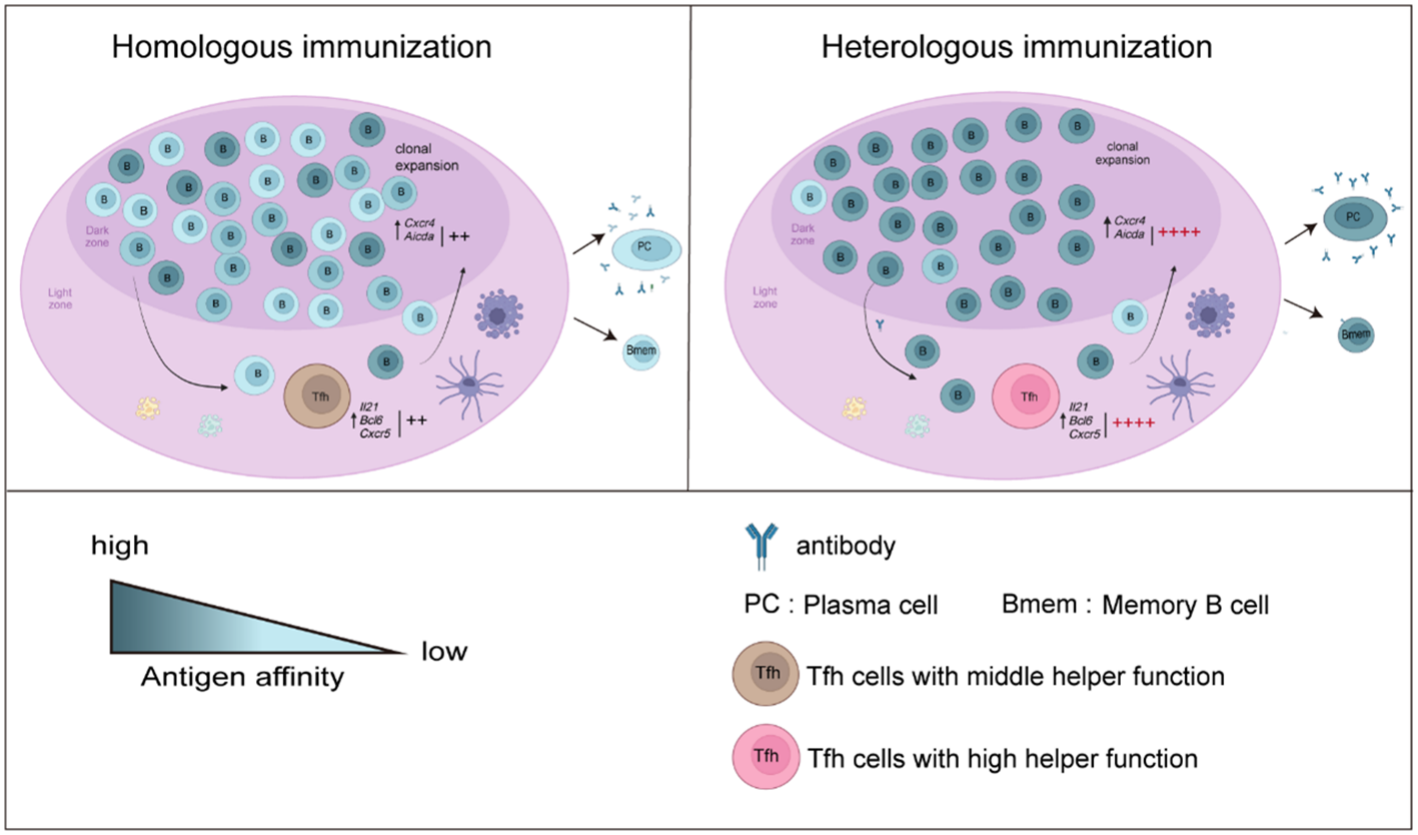
Overview of the results in this study. B-cell responses and Tfh cell function increased after a heterologous booster dose. The proliferation and helper function of Tfh cells in the heterologous group were improved. The expression of *Il21*, *Bcl6* and *Cxcr5* was elevated. A heterologous booster dose promoted B-cell activation and a robust antibody response. These B cells displayed increased SHMs, which were enriched mainly in S1- and S2-specific plasma cells. Additionally, heterologous vaccination increased the expansion of B cells specific to the conserved regions in the spike protein.

In our study, to assess the mechanism by which heterologous booster vaccination enhances antibody responses, Tfh and B-cell responses in mice were compared. We found that the frequencies and helper function of Tfh cells were enhanced after a heterologous booster dose according to the *in vitro* coculture experiment. The expression of genes such as *Il21*, *Bcl6* and *Cxcr5*, which regulate the function and proliferation of Tfh cells, was upregulated. Moreover, we found that the heterologous treatment increased the frequencies, activation and antibody production ability of B cells. These B cells showed increased expression of *Aicda*. Libra-seq showed that a heterologous booster dose induced increased SHMs in S1-, S2- and NTD-specific B cells, which were mainly enriched in S1- and S2-specific plasma cells. Additionally, clonal expansion of B cells specific to conserved regions in the spike protein was found in the heterologous group. In summary, the percentages and functions of Tfh and B cells improved after heterologous immunization, which may induce more robust antibody responses (**Figure 6**).

Tfh cells provide cytokines and costimulation signals to B cells in the process of GC activation (17, 18, 39, 40). The ability of Tfh cells to activate B cells depends on the population and function of the cells (41–43). We found that Tfh cells expanded after a heterologous booster dose, indicating that a heterologous strategy may promote additional Tfh cells to provide costimulation signals for B cells. The transcription factor BCL-6 promotes the expression of CXCR5 in CD4+ T cells, and CXCR5 promotes the migration of differentiating Tfh cells to the T-B border by binding to CXCL13 (40, 44). Interlink 21 (IL-21) regulates Tfh cell proliferation and differentiation, and Tfh cells can in turn stimulate the proliferation of surrounding Tfh cells through the paracrine cytokine IL-21 (45). We assessed *Il21* and *Bcl6* mRNA expression and found that they were increased two- to three-fold after immunization with the adenovirus vector. These findings suggest that increased expression of these genes in heterologous strategies may promote Tfh differentiation and increase Tfh cell populations.

*In vitro* coculture experiments revealed that the Tfh cells derived from heterologously vaccinated mice were able to activate more B cells to secrete higher antibody titers. These results suggest that a heterologous vaccination strategy may enhance Tfh cell helper function. It has been shown that BCL-6 expressed by Tfh cells enhances B-cell activation by upregulating CD40L expression, an activation signal that provides a costimulation signal for B cells in the GC (46). In our study, we found that *Bcl6* expression in Tfh cells was elevated after a heterologous booster dose, which may provide additional activation signals for B cells. Although Il21 and Bcl6 were upregulated in the heterologous group, their detailed regulatory process deserves further study.

GCs are considered highly competitive environments where B cells initially encoding polyreactive, low-affinity antibodies evolve via iterative rounds of SHM and selection into clones expressing high-affinity BCRs (34, 43, 47–50). The highest affinity BCRs differentiate into plasma cells, which are the main cells that secrete antibodies (51). Cells expressing low-affinity BCRs differentiate into memory B cells, thereby supporting diversity (48–50). To assess SHM in the heterologous group, spike-, S1-, S2- and NTD-specific B cells were analyzed. The heterologous group exhibited more SHMs than the homologous group in terms of CD19+ B cells. Interestingly, the number of spike-, S1- and S2-specific plasma cells increased 4- to 6-fold in response to the heterologous strategy, which may be beneficial for obtaining high-affinity plasma cells and elevated antibody titers. Moreover, it has been shown that memory B-cell SHMs play an important role in the long-term protection ability of SARS-CoV-2 vaccines (52). According to our results, the number of SHMs in the memory B cells of the heterologous group was higher than that in the homologous group, suggesting that a heterologous booster dose may have a better long-term protective effect.

SHM and proliferation occur in the CXCR4^hi^ B cells sited in the DZ (53). To locate in this area, B cells increase the expression of CXCR4, which can interact with CXCL12 on reticular cells, facilitating B cell migration from LZ to DZ. Our study revealed a significant upregulation of genes associated with cell migration, such as *Cxcr4* and *Cxcl12*, following a heterologous booster dose as determined by RNA-seq analysis. The migration to DZ and the SHM process can further boost the screening B cells clones producing higher affinity antibodies. SHM is induced by AID, which is expressed predominantly in activated mature B cells and targets all transcriptionally active genes to catalyze the mismatch of U:G variable regions (54–56). In our study, we found elevated expression of *Aicda* (encoded AID) at the gene level. Additionally, SHMs in antigen-specific B cells were observed via Libra-seq in the heterologous vaccination group. Moreover, AID induces point mutations in conserved regions of immunoglobulin DNA, base excision repair (BER) initiates, and a DNA double-strand break occurs. The C region of IgM/IgD is replaced by the C gene downstream of the gene chain to produce the IgG, IgE and IgA subtypes (55).

SARS-CoV-2 S protein is present on the surface of virions in the trimeric form, which consists of S1 subunit encompassing the NTD and the receptor-binding domain (RBD); and a membrane-proximal S2 subunit which is responsible for fusion of viral and cellular membranes (57). Effective NAb against SARS-CoV-2 primarily target the RBD, which is responsible for binding to the angiotensin-converting enzyme 2 (ACE2) on host (57). However, Chi et al. reported that the NTD-specific monoclonal antibody 4A8, isolated from COVID-19 recovered patients, exhibited a high neutralizing ability against authentic SARS-CoV-2 (51). In addition to S1, Kim and his co-workers demonstrated that mice immunized with the S2 protein could induce NAb against pseudoviral SARS-CoV-2 (58). In our homologous vaccination group, although the S1-specific antibody titer did not increase after the 4th or 5th dose, the IgG titers specific to S2 and NTD continuously increased, which is similar to that of other studies (59). Moreover, we observed an increase in IgG titers and expansion of B-cell clones specific to S2 and NTD in heterologous group, suggesting that a heterologous booster dose may enhance immune responses against these proteins and contribute to the overall production of NAb.

In our study, differences in Tfh and B-cell responses after homologous and heterologous booster vaccinations were compared, and the mechanism of increased antibody responses after a heterologous booster dose was elucidated. Adenovirus (Ad) is a nonenveloped virus with a linear double-stranded DNA (dsDNA) genome, which is recognized by TLR9 in plasmacytoid DCs and promotes type I interferon production (60). Then, the innate immune responses initiate (60, 61). In inactivated vaccines, aluminum adjuvants promote IL-1β production and the formation of the NLRP3/ASC1/NLR complex, which can activate the NF-kB signaling pathway. Through this process, TLR-independent and CD4+ T-cell-dependent responses are initiated (61–63). Additionally, the processes of uptake, processing and presentation of targeted antigens also differ between these two types of vaccines. For example, spike protein coded by dsDNA is primarily produced and presented to CD8+T cells by MHC-I through the endogenous pathway. In contrast, responses to inactivated vaccines result in antigens being mainly presented to CD4+T cells via MHC-II through the exogenous presentation pathway. The activated CD4+T and CD8+T cells secrete different cytokines, potentially leading to the discrepancies of Th biases and B-cell responses. Therefore, we believe that the prime immunization with inactivated vaccines followed by adenovirus vaccines boost may initiate comprehensive or complementary immune responses, leading to higher antibody production (64, 65). Therefore, we believe that the prime immunization with inactivated vaccines followed by adenovirus vaccines boost may initiate comprehensive or complementary immune responses, leading to higher antibody production. However, whether other types of heterologous vaccination regimes can induce similar results deserves further study, since antibody induction is a complex process regulated by multiple types of cells, cytokines, and signaling pathways when antigens encounter immune system. In addition to germinal center Tfh and B-cell responses focused in this study, other crucial elements, such as antigen concentration and distribution routes can also affect the immune system to different extents.

In summary, Tfh and B-cell responses to heterologous and homologous vaccination were compared to explore the mechanism of increased antibody responses to heterologous booster doses. This study may provide a reference for optimizing vaccine strategies.

## 5 Conclusion

In our study, Tfh and B-cell responses in GC after homologous inactivated vaccination and heterologous prime boosting vaccination with adenoviral vaccines were analyzed to investigate how these two strategies regulate antibody responses. The results showed that populations and the helper function of Tfh cells were enhanced with the heterologous strategy. This improvement may be induced by increased gene expression of *Il21, Bcl6* and *Cxcr5* in Tfh cells. In the heterologous group, B cells exhibited more robust activation and a higher ability to produce antibodies, along with increased SHMs in S2- and NTD-specific plasma cells. Additionally, heterologous vaccination led to an expansion of B cell specific to conserved regions in the spike protein. These may contribute to increased antibody titers by a heterologous booster dose.

## Data availability statement

The datasets presented in this study can be found in online repositories. The names of the repository/repositories and accession number(s) can be found below: NCBI via accession ID PRJNA1111280.

## Ethics statement

The animal study was approved by Institutional Animal Care and Use Committee (IACUC) at the Beijing Institute of Biological Products Co., Ltd. The study was conducted in accordance with the local legislation and institutional requirements.

## Author contributions

YaS: Writing – original draft. JW: Writing – original draft. ZY: Writing – original draft. QH: Writing – original draft, Funding acquisition. CB: Writing – original draft. YX: Writing – original draft. YuS: Writing – original draft. SL: Writing – original draft. YQ: Writing – original draft. HY: Writing – review & editing. CL: Writing – review & editing.

## References

[1] Atmar RL Lyke KE Deming ME Jackson LA Branche AR El Sahly HM. Homologous and heterologous Covid-19 booster vaccinations. N Engl J Med. (2022) 386:NEJMoa2116414. doi: 10.1056/NEJMoa2116414 35081293 PMC8820244

[2] Mallah SI Alawadhi A Jawad J Wasif P Alsaffar B Alalawi E. Safety and efficacy of COVID-19 prime-boost vaccinations: Homologous BBIBP-CorV versus heterologous BNT162b2 boosters in BBIBP-CorV-primed individuals. Vaccine. (2023) 41:1925–33. doi: 10.1016/j.vaccine.2023.01.032 PMC9868355 36725431

[3] Nordström P Ballin M Nordström A. Effectiveness of heterologous ChAdOx1 nCoV-19 and mRNA prime-boost vaccination against symptomatic Covid-19 infection in Sweden: A nationwide cohort study. Lancet Reg Health Eur. (2021) 11:100249. doi: 10.1016/j.lanepe.2021.100249 34693387 PMC8520818

[4] Pozzetto B Legros V Djebali S Barateau V Guibert N Villard M. Immunogenicity and efficacy of heterologous ChAdOx1-BNT162b2 vaccination. Nature. (2021) 600:701–6. doi: 10.1038/s41586-021-04120-y 34673755

[5] Zhang X Xia J Jin L Wu Y Zheng X Cao X. Effectiveness of homologous or heterologous immunization regimens against SARS-CoV-2 after two doses of inactivated COVID-19 vaccine: A systematic review and meta-analysis. Hum Vaccin Immunother. (2023) 19:2221146. doi: 10.1080/21645515.2023.2221146 37344370 PMC10288895

[6] Jara A Undurraga EA Zubizarreta JR González C Pizarro A Acevedo J. Effectiveness of homologous and heterologous booster doses for an inactivated SARS-CoV-2 vaccine: a large-scale prospective cohort study. Lancet Global Health. (2022) 10:e798–806. doi: 10.1016/S2214-109X(22)00112-7 PMC9034854 35472300

[7] Li J-X Wu S-P Guo X-L Tang R Huang B-Y Chen X-Q. Safety and immunogenicity of heterologous boost immunisation with an orally administered aerosolised Ad5-nCoV after two-dose priming with an inactivated SARS-CoV-2 vaccine in Chinese adults: a randomised, open-label, single-centre trial. Lancet Respir Med. (2022) 10:739–48. doi: 10.1016/S2213-2600(22)00087-X PMC9122540 35605625

[8] He Q Mao Q An C Zhang J Gao F Bian L. Heterologous prime-boost: breaking the protective immune response bottleneck of COVID-19 vaccine candidates. Emerging Microbes Infections. (2021) 10:629–37. doi: 10.1080/22221751.2021.1902245 PMC8009122 33691606

[9] Spencer AJ McKay PF Belij-Rammerstorfer S Ulaszewska M Bissett CD Hu K. Heterologous vaccination regimens with self-amplifying RNA and adenoviral COVID vaccines induce robust immune responses in mice. Nat Commun. (2021) 12:2893. doi: 10.1038/s41467-021-23173-1 34001897 PMC8129084

[10] Hu SL Abrams K Barber GN Moran P Zarling JM Langlois AJ. Protection of macaques against SIV infection by subunit vaccines of SIV envelope glycoprotein gp160. Science. (1992) 255:456–9. doi: 10.1126/science.1531159 1531159

[11] Liu Z Lu L Jiang S. Application of “B+1” heterologous boosting strategy for preventing infection of SARS-CoV-2 variants with resistance to broad-spectrum coronavirus vaccines. Emerg Microbes Infect. (2023) 12:2192817. doi: 10.1080/22221751.2023.2192817 36927258 PMC10071895

[12] He R Zheng X Zhang J Liu B Wang Q Wu Q. SARS-CoV-2 spike-specific TFH cells exhibit unique responses in infected and vaccinated individuals. Signal Transduct Target Ther. (2023) 8:393. doi: 10.1038/s41392-023-01650-x 37802996 PMC10558553

[13] Zou P Zhang P Deng Q Wang C Luo S Zhang L. Two novel adenovirus vectors mediated differential antibody responses via interferon-α and natural killer cells. Microbiol Spectr. (2023) 11:e0088023. doi: 10.1128/spectrum.00880-23 37347197 PMC10434031

[14] Gao F-X Wu R-X Shen M-Y Huang J-J Li T-T Hu C. Extended SARS-CoV-2 RBD booster vaccination induces humoral and cellular immune tolerance in mice. iScience. (2022) 25:105479. doi: 10.1016/j.isci.2022.105479 36338436 PMC9625849

[15] Tani Y Takita M Wakui M Saito H Nishiuchi T Zhao T. Five doses of the mRNA vaccination potentially suppress ancestral-strain stimulated SARS-CoV2-specific cellular immunity: a cohort study from the Fukushima vaccination community survey, Japan. Front Immunol. (2023) 14:1240425. doi: 10.3389/fimmu.2023.1240425 37662950 PMC10469480

[16] Li X Zeng F Yue R Ma D Meng Z Li Q. Heterologous booster immunization based on inactivated SARS-CoV-2 vaccine enhances humoral immunity and promotes BCR repertoire development. Vaccines. (2024) 12:120. doi: 10.3390/vaccines12020120 38400104 PMC10891849

[17] Sheikh AA Groom JR. Transcription tipping points for T follicular helper cell and T-helper 1 cell fate commitment. Cell Mol Immunol. (2021) 18:528–38. doi: 10.1038/s41423-020-00554-y PMC7525231 32999454

[18] Cui D Tang Y Jiang Q Jiang D Zhang Y Lv Y. Follicular helper T cells in the immunopathogenesis of SARS-CoV-2 infection. Front Immunol. (2021) 12:731100. doi: 10.3389/fimmu.2021.731100 34603308 PMC8481693

[19] Yang H Xie Y Lu S Sun Y Wang K Li S. Independent protection and influence of the spike-specific antibody response of SARS-CoV-2 nucleocapsid protein (N) in whole-virion vaccines. Vaccines (Basel). (2023) 11:1681. doi: 10.3390/vaccines11111681 38006013 PMC10675215

[20] Zhang Y Zeng G Pan H Li C Hu Y Chu K. Safety, tolerability, and immunogenicity of an inactivated SARS-CoV-2 vaccine in healthy adults aged 18–59 years: a randomised, double-blind, placebo-controlled, phase 1/2 clinical trial. Lancet Infect Dis. (2021) 21:181–92. doi: 10.1016/S1473-3099(20)30843-4 PMC7832443 33217362

[21] Wang W Li Y Hao J He Y Dong X Fu Y-X. The interaction between lymphoid tissue inducer-like cells and T cells in the mesenteric lymph node restrains intestinal humoral immunity. Cell Rep. (2020) 32:107936. doi: 10.1016/j.celrep.2020.107936 32698011

[22] Song I-W Nagamani SCS Nguyen D Grafe I Sutton VR Gannon FH. Targeting TGF-β for treatment of osteogenesis imperfecta. J Clin Invest. (2022) 132:e152571. doi: 10.1172/JCI152571 35113812 PMC8970679

[23] Zhao Z Xu B Wang S Zhou M Huang Y Guo C. Tfh cells with NLRP3 inflammasome activation are essential for high-affinity antibody generation, germinal centre formation and autoimmunity. Ann Rheum Dis. (2022) 81:1006–12. doi: 10.1136/annrheumdis-2021-221985 PMC9262832 35414518

[24] Coquery CM Loo WM Wade NS Bederman AG Tung KS Lewis JE. BAFF regulates T follicular helper cells and affects accumulation and IFNγ production in autoimmunity. Arthritis Rheumatol. (2015) 67:773–84. doi: 10.1002/art.38950 PMC4342294 25385309

[25] Nurieva RI Chung Y Hwang D Yang XO Kang HS Ma L. Generation of T follicular helper cells is mediated by interleukin-21 but independent of T helper 1, 2, or 17 cell lineages. Immunity. (2008) 29:138–49. doi: 10.1016/j.immuni.2008.05.009 PMC2556461 18599325

[26] Zhao H Yang J Qian Q Wu M Li M Xu W. Mesenteric CD103+DCs initiate switched coxsackievirus B3 VP1-specific IgA response to intranasal chitosan-DNA vaccine through secreting BAFF/IL-6 and promoting Th17/Tfh differentiation. Front Immunol. (2018) 9:2986. doi: 10.3389/fimmu.2018.02986 30619341 PMC6305319

[27] Garg R Theaker M Martinez EC Drunen Little Hurk S. A single intranasal immunization with a subunit vaccine formulation induces higher mucosal IgA production than live respiratory syncytial virus. Virology. (2016) 499:288–97. doi: 10.1016/j.virol.2016.09.023 27721128

[28] Dominguez-Sola D Kung J Holmes AB Wells VA Mo T Basso K. The FOXO1 transcription factor instructs the germinal center dark zone program. Immunity. (2015) 43:1064–74. doi: 10.1016/j.immuni.2015.10.015 26620759

[29] Weskamm LM Dahlke C Addo MM. Flow cytometric protocol to characterize human memory B cells directed against SARS-CoV-2 spike protein antigens. STAR Protoc. (2022) 3:101902. doi: 10.1016/j.xpro.2022.101902 36595922 PMC9663734

[30] Pušnik J Richter E Schulte B Dolscheid-Pommerich R Bode C Putensen C. Memory B cells targeting SARS-CoV-2 spike protein and their dependence on CD4+ T cell help. Cell Rep. (2021) 35:109320. doi: 10.1016/j.celrep.2021.109320 34146478 PMC8192958

[31] Nuñez G Hockenbery D McDonnell TJ Sorensen CM Korsmeyer SJ. Bcl-2 maintains B cell memory. Nature. (1991) 353:71–3. doi: 10.1038/353071a0 1908951

[32] Yu S-C Chen K-C Huang RY-J. Nodal reactive proliferation of monocytoid B-cells may represent atypical memory B-cells. J Microbiol Immunol Infect. (2023) 56:729–38. doi: 10.1016/j.jmii.2023.03.010 37080839

[33] Ansari A Sachan S Jit BP Sharma A Coshic P Sette A. An efficient immunoassay for the B cell help function of SARS-CoV-2-specific memory CD4+ T cells. Cell Rep Methods. (2022) 2:100224. doi: 10.1016/j.crmeth.2022.100224 35571764 PMC9085463

[34] Young C Brink R. The unique biology of germinal center B cells. Immunity. (2021) 54:1652–64. doi: 10.1016/j.immuni.2021.07.015 34380063

[35] Buckner CM Kardava L El Merhebi O Narpala SR Serebryannyy L Lin BC. Interval between prior SARS-CoV-2 infection and booster vaccination impacts magnitude and quality of antibody and B cell responses. Cell. (2022) 185:4333–46.e14. doi: 10.1016/j.cell.2022.09.032 36257313 PMC9513331

[36] Park S-R. Activation-induced cytidine deaminase in B cell immunity and cancers. Immune Netw. (2012) 12:230–9. doi: 10.4110/in.2012.12.6.230 PMC3566417 23396757

[37] Khurana S Frasca D Blomberg B Golding H. AID activity in B cells strongly correlates with polyclonal antibody affinity maturation *in-vivo* following pandemic 2009-H1N1 vaccination in humans. PloS Pathog. (2012) 8:e1002920. doi: 10.1371/journal.ppat.1002920 23028320 PMC3441753

[38] Yewdell WT Kim Y Chowdhury P Lau CM Smolkin RM Belcheva KT. A hyper-IgM syndrome mutation in activation-induced cytidine deaminase disrupts G-quadruplex binding and genome-wide chromatin localization. Immunity. (2020) 53:952–70.e11. doi: 10.1016/j.immuni.2020.10.003 33098766 PMC8356774

[39] Karnowski A Chevrier S Belz GT Mount A Emslie D D’Costa K. B and T cells collaborate in antiviral responses via IL-6, IL-21, and transcriptional activator and coactivator, Oct2 and OBF-1. J Exp Med. (2012) 209:2049–64. doi: 10.1084/jem.20111504 PMC3478936 23045607

[40] Crotty S. T follicular helper cell biology: A decade of discovery and diseases. Immunity. (2019) 50:1132–48. doi: 10.1016/j.immuni.2019.04.011 PMC6532429 31117010

[41] Shlomchik MJ Weisel F. Germinal center selection and the development of memory B and plasma cells: Germinal center differentiation and selection. Immunol Rev. (2012) 247:52–63. doi: 10.1111/j.1600-065X.2012.01124.x 22500831

[42] Allen CDC Okada T Cyster JG. Germinal-center organization and cellular dynamics. Immunity. (2007) 27:190–202. doi: 10.1016/j.immuni.2007.07.009 17723214 PMC2242846

[43] Cavazzoni CB Hanson BL Podestà MA Bechu ED Clement RL Zhang H. Follicular T cells optimize the germinal center response to SARS-CoV-2 protein vaccination in mice. Cell Rep. (2022) 38:110399. doi: 10.1016/j.celrep.2022.110399 35139367 PMC8806144

[44] Kaneko N Kuo H-H Boucau J Farmer JR Allard-Chamard H Mahajan VS. Loss of Bcl-6-expressing T follicular helper cells and germinal centers in COVID-19. Cell. (2020) 183:143–57.e13. doi: 10.1016/j.cell.2020.08.025 32877699 PMC7437499

[45] Fallet B Hao Y Florova M Cornille K de Los Aires AV Girelli Zubani G. Chronic viral infection promotes efficient germinal center B cell responses. Cell Rep. (2020) 30:1013–26.e7. doi: 10.1016/j.celrep.2019.12.023 31995746 PMC6996002

[46] Liu D Yan J Sun J Liu B Ma W Li Y. BCL6 controls contact-dependent help delivery during follicular T-B cell interactions. Immunity. (2021) 54:2245–55.e4. doi: 10.1016/j.immuni.2021.08.003 34464595 PMC8528402

[47] Nutt SL Hodgkin PD Tarlinton DM Corcoran LM. The generation of antibody-secreting plasma cells. Nat Rev Immunol. (2015) 15:160–71. doi: 10.1038/nri3795 25698678

[48] Zhang Y Garcia-Ibanez L Ulbricht C Lok LSC Pike JA Mueller-Winkler J. Recycling of memory B cells between germinal center and lymph node subcapsular sinus supports affinity maturation to antigenic drift. Nat Commun. (2022) 13:2460. doi: 10.1038/s41467-022-29978-y 35513371 PMC9072412

[49] Viant C Wirthmiller T ElTanbouly MA Chen ST Cipolla M Ramos V. Germinal center–dependent and –independent memory B cells produced throughout the immune response. J Exp Med. (2021) 218:e20202489. doi: 10.1084/jem.20202489 34106207 PMC8193567

[50] Sprumont A Rodrigues A McGowan SJ Bannard C Bannard O. Germinal centers output clonally diverse plasma cell populations expressing high- and low-affinity antibodies. Cell. (2023) 186:5486–99.e13. doi: 10.1016/j.cell.2023.10.022 37951212 PMC7617393

[51] Chi X Yan R Zhang J Zhang G Zhang Y Hao M. A neutralizing human antibody binds to the N-terminal domain of the Spike protein of SARS-CoV-2. Science. (2020) 369:650–5. doi: 10.1126/science.abc6952 PMC7319273 32571838

[52] Ai J Guo J Zhang H Zhang Y Yang H Lin K. Cellular basis of enhanced humoral immunity to SARS-CoV-2 upon homologous or heterologous booster vaccination analyzed by single-cell immune profiling. Cell Discovery. (2022) 8:114. doi: 10.1038/s41421-022-00480-5 36270988 PMC9587260

[53] Sander S Chu VT Yasuda T Franklin A Graf R Calado DP. PI3 kinase and FOXO1 transcription factor activity differentially control B cells in the germinal center light and dark zones. Immunity. (2015) 43:1075–86. doi: 10.1016/j.immuni.2015.10.021 26620760

[54] Xie X Gan T Rao B Zhang W Panchakshari RA Yang D. C-terminal deletion-induced condensation sequesters AID from IgH targets in immunodeficiency. EMBO J. (2022) 41:e109324. doi: 10.15252/embj.2021109324 35471583 PMC9156971

[55] Delgado P Álvarez-Prado ÁF Marina-Zárate E Sernandez IV Mur SM de la Barrera J. Interplay between UNG and AID governs intratumoral heterogeneity in mature B cell lymphoma. PloS Genet. (2020) 16:e1008960. doi: 10.1371/journal.pgen.1008960 33362210 PMC7790409

[56] Yu K. AID function in somatic hypermutation and class switch recombination. Acta Biochim Biophys Sin (Shanghai). (2022) 54:759–66. doi: 10.3724/abbs.2022070 PMC9827813 35975606

[57] Hajizadeh F Khanizadeh S Khodadadi H Mokhayeri Y Ajorloo M Malekshahi A. SARS-COV-2 RBD (Receptor binding domain) mutations and variants (A sectional-analytical study). Microbial Pathogenesis. (2022) 168:105595. doi: 10.1016/j.micpath.2022.105595 35597364 PMC9116045

[58] Kim K-H Bhatnagar N Jeeva S Oh J Park BR Shin CH. Immunogenicity and neutralizing activity comparison of SARS-CoV-2 spike full-length and subunit domain proteins in young adult and old-aged mice. Vaccines. (2021) 9:316. doi: 10.3390/vaccines9040316 33805473 PMC8066235

[59] Wang J Deng C Liu M Liu Y Li L Huang Z. A fourth dose of the inactivated SARS-CoV-2 vaccine redistributes humoral immunity to the N-terminal domain. Nat Commun. (2022) 13:6866. doi: 10.1038/s41467-022-34633-7 36369243 PMC9651894

[60] Di Paolo NC Miao EA Iwakura Y Murali-Krishna K Aderem A Flavell RA. Virus binding to a plasma membrane receptor triggers interleukin-1 alpha-mediated proinflammatory macrophage response in *vivo* . Immunity. (2009) 31:110–21. doi: 10.1016/j.immuni.2009.04.015 PMC2759279 19576795

[61] Teijaro JR Farber DL. COVID-19 vaccines: modes of immune activation and future challenges. Nat Rev Immunol. (2021) 21:195–7. doi: 10.1038/s41577-021-00526-x PMC7934118 33674759

[62] Sprent J King C. COVID-19 vaccine side effects: The positives about feeling bad. Sci Immunol. (2021) 6:eabj9256. doi: 10.1126/sciimmunol.abj9256 34158390 PMC9267253

[63] Mettelman RC Allen EK Thomas PG. Mucosal immune responses to infection and vaccination in the respiratory tract. Immunity. (2022) 55:749–80. doi: 10.1016/j.immuni.2022.04.013 PMC9087965 35545027

[64] Strawbridge AB Blum JS. Autophagy in MHC class II antigen processing. Curr Opin Immunol. (2007) 19:87–92. doi: 10.1016/j.coi.2006.11.009 17129719

[65] English L Chemali M Duron J Rondeau C Laplante A Gingras D. Autophagy enhances the presentation of endogenous viral antigens on MHC class I molecules during HSV-1 infection. Nat Immunol. (2009) 10:480–7. doi: 10.1038/ni.1720 PMC3885169 19305394

