# Supplementary material for: Heterologous booster vaccination enhances antibody responses to SARS-CoV-2 by improving Tfh function and increasing B-cell clonotype SHM frequency
Source: Front Immunol. 2024 Jun 21;15:1406138. doi: 10.3389/fimmu.2024.1406138 (PMC11224535; doi:10.3389/fimmu.2024.1406138)
Supplement: Supplementary file 1 [file DataSheet_1.docx]

Supplementary Material

Article Title

Yanli Song^1^, Jiaolei Wang^2^, Zhihui Yang^1^, Qian He^2^, Chunting Bao^2^, Ying Xie^2^, Yufang Sun^2^, Shuyan Li^2^, Yaru Quan^2^, Huijie Yang^2*^ and Changgui Li^2*^

^1^ Divsion of the second Vaccines, Wuhan Institute of Biological Products Co. Ltd., Beijing, China

^2^Divsion of Respiratory Virus Vaccines, National Institutes for Food and Drug Control, Beijing, China

*** Correspondence:**Huijie Yang✉[jieer6423@outlook.com](mailto:jieer6423@outlook.com), Changgui Li✉changguili@aliyun.com

## 1 Supplementary Data Legends

Supplementary Figure 1. Antibody response induced by 5 homologous doses of an inactivated SARS-CoV-2 vaccine in mice.

Supplementary Figure 2. Correlations between GC B cells and antibody titers in heterologous and homologous groups.

Supplementary Figure 3. Additional characterization of somatic hypermutation (SHM) and clonal expansion in heterologous groups.

## 2 Supplementary Figures

## Supplementary Figure 1. Antibody response induced by 5 homologous doses of an inactivated SARS-CoV-2 vaccine in mice


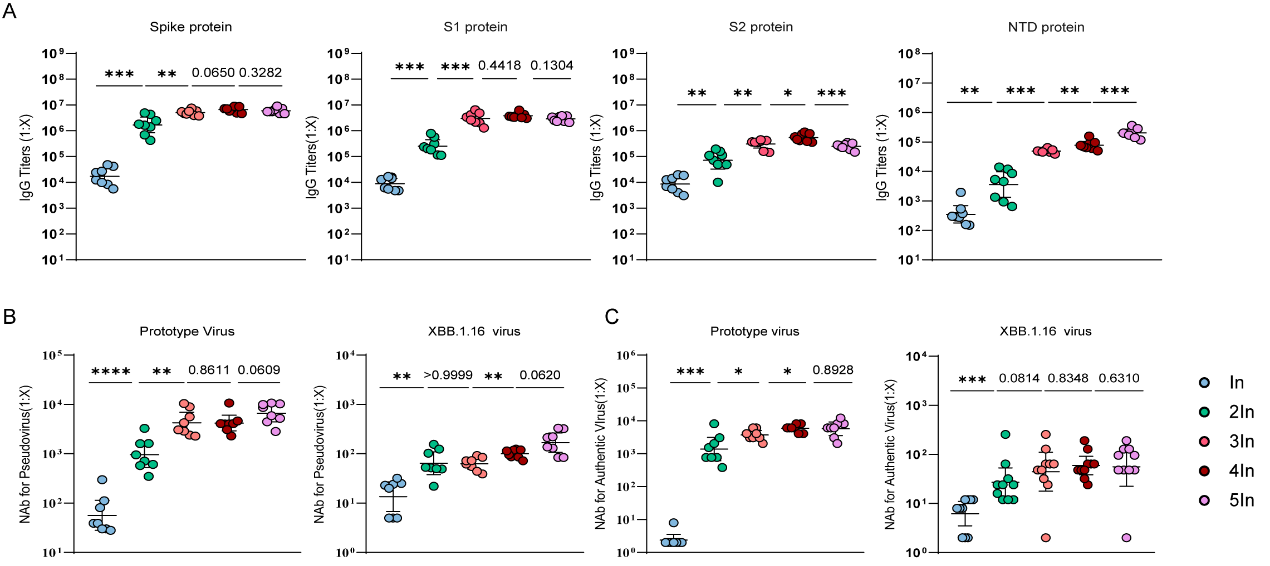


(A) Spike-, S1-, S2-, and NTD-specific IgG titers. NAb titers against (**B**) prototyped and XBB.1.16 pseudovirus and (C) authentic prototyped and XBB.1.16 virus. n=8 per vaccination group. Each symbol represents an individual animal. In, 2In, 3In, 4In, and 5In represent mice vaccinated with inactivated vaccines for one to five doses. The data are presented as the geometric mean ± SEM. The Mann‒Whitney U test was used to analyze the differences between the indicated groups. *p < 0.05, **p < 0.01, and ***p < 0.001 were considered to indicate two-tailed significant differences. The numbers in the graph represent p values.

## Supplementary Figure 2. Correlations between GC B cells and antibody titers in heterologous and homologous groups


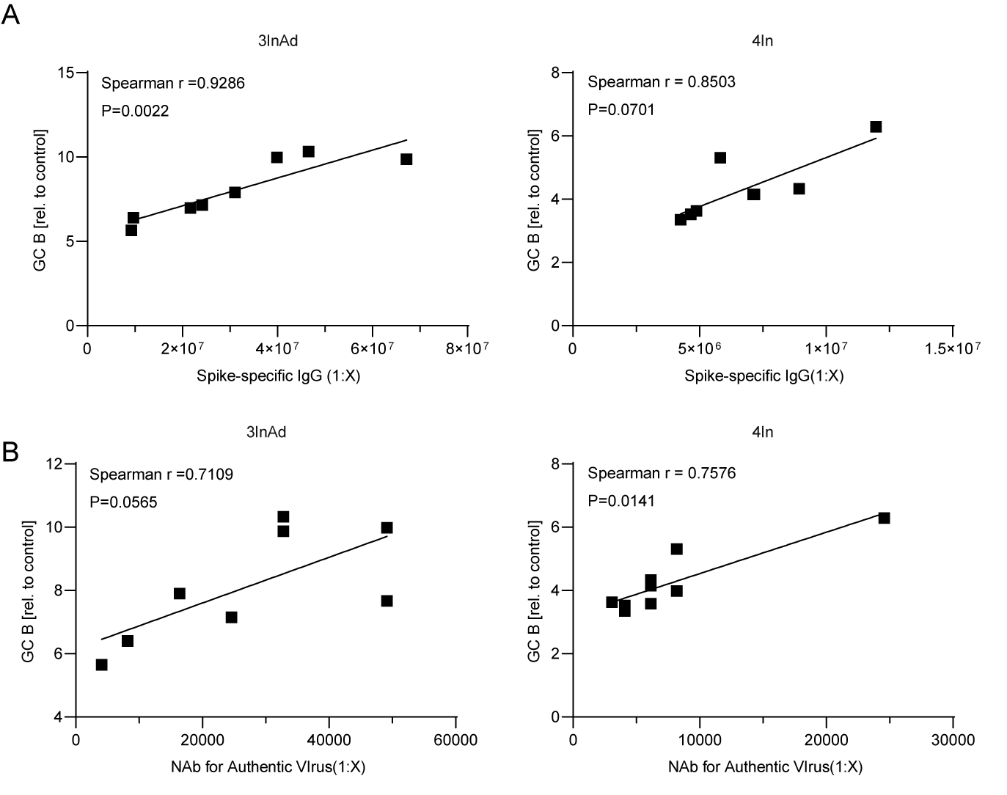


Spearman’s rank correlation coefficient was used to describe the association between the frequencies of GC B cells and (A) spike-specific IgG titer or (B) NAb against the authentic virus. p < 0.05 was considered to indicate a two-tailed significant difference. n=8 in each vaccinated group; n=6 in the PBS group.

## Supplementary Figure 3. Additional characterization of somatic hypermutation (SHM) and clonal expansion in heterologous groups


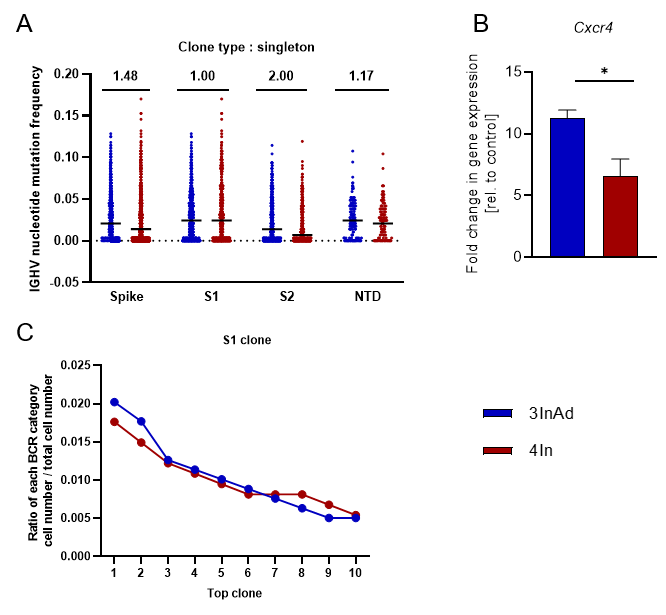


Mutation frequency for spike-, S1-, S2- and NTD-specific (A) singletons. (B) *Cxcr4* mRNA expression in the homologous group. (C) The ratio of the cell number in each BCR classification to the S1-specific B-cell number in the top 10 BCRs. Ratio=cell count of each BCR classification/antigen-specific B-cell count. (A) Each dot represents the hypermutation frequency in one B cell. Ratio=3InAd mutation ratio/4In mutation ratio. (B) n=8 in the SARS-CoV-2 vaccination group and n=6 in the PBS group. One sequenced sample was formed by mixing 10 mouse spleen cells in each group. The Mann‒Whitney U test was used to analyze the differences between the indicated groups. *p < 0.05 was considered to indicate a two-tailed significant difference.
